# Unveiling immunogenic characteristics and neoantigens in endometrial cancer with POLE hotspot mutations for improved immunotherapy

**DOI:** 10.3389/fimmu.2025.1528532

**Published:** 2025-01-27

**Authors:** Jian Huang, Shuangna Song, Yihua Yin, Yinyan He, Huimin Wang, Ye Gu, Laman He, Xintao Wang, Xiaocao Miao, Zhigang Zhang, Xueli Zhang, Yiran Li

**Affiliations:** ^1^ Shanghai Key Laboratory of Maternal Fetal Medicine, Shanghai Institute of Maternal-Fetal Medicine and Gynecologic Oncology, Shanghai First Maternity and Infant Hospital, School of Medicine, Tongji University, Shanghai, China; ^2^ Department of Gynecology, Shanghai First Maternity and Infant Hospital, Tongji University School of Medicine, Shanghai, China; ^3^ State Key Laboratory of Systems Medicine for Cancer, Shanghai Cancer Institute, Ren Ji Hospital, School of Medicine, Shanghai Jiao Tong University, Shanghai, China; ^4^ Center for Reproductive Medicine, Shanghai First Maternity and Infant Hospital, School of Medicine, Tongji University, Shanghai, China

**Keywords:** endometrial cancer (EC), POLE, tumor neoantigens, immunotherapy, CD8^+^ T cells

## Abstract

**Background:**

Immunotherapy, especially with the use of immune checkpoint inhibitors, has demonstrated efficacy for a variety of malignant tumors. However, the potential of immunotherapy for endometrial cancer (EC) with POLE mutations remains underexplored.

**Methods:**

We utilized multiple databases and clinical specimens to investigate the immunogenicity profiles of EC patients carrying POLE mutations. One particular hotspot mutation POLE^P286R^ was identified and further studied. Consequently, by constructing human leukocyte antigen (HLA) tetramers and incubating them with patients’ peripheral blood mononuclear cells (PBMCs), T cells capable of recognizing the POLE^P286R^ mutation were sorted for further transcriptomic, proteomic and T-cell receptor (TCR) sequencing analyses and for an organoid EC model.

**Results:**

Tumor- and immune-related pathways were shown to be activated in the POLE^P286R^ mutant group. Importantly, by using an organoid model of EC, we further confirmed the antitumor potential of T cells that were specific to the POLE^P286R^ mutation.

**Conclusions:**

Our study uncovers the pronounced immunogenicity of POLE-mutant EC and characterizes neoantigens that are unique to the POLE^P286R^ mutation, thus providing a promising new immunotherapeutic strategy for EC.

## Introduction

EC is the sixth most common cancer in women and account for 7% of all malignant tumors (1). In recent years, with changes in the socioeconomic structure, diet and lifestyle habits, the incidence of EC has increased, and the average age of EC occurrence has decreased (2, 3). Although early diagnosis, surgical treatment and chemotherapy have reduced the mortality rate of EC to some extent (4), treatment options are very limited for some young patients who need to preserve their reproductive function. For patients with advanced or recurrent EC in particular, the clinical prognosis remains poor (5). Immunotherapies for EC started late, and most clinical studies have focused on EC with microsatellite instability, while immunotherapies for POLE-mutant EC have not yet been studied in depth (6, 7). Therefore, individualized and precise treatment strategies for EC are urgently needed.

In 2013, The Cancer Genome Atlas (TCGA) identified four categories of endometrial carcinomas based on their molecular features: ultra-mutated (with mutations in the polymerase ϵ (POLE) gene) tumors, tumors with microsatellite instability (MSI), copy number low/microsatellite stable (CNL) tumors and copy number high (CNH) tumors (8). Among the four molecular typologies defined by the TCGA, the POLE hypermutant phenotype is one of the specific and recognized molecular types. POLE mutant ECs usually present with early lesions, high tumor grade, but showed a better prognosis, which is closely associated with the immune response, which warrants further novel immunotherapeutic explorations for POLE mutant EC (9, 10). In addition, a recent real-world cohort study from the United Kingdom assessed the clinical and genomic characteristics of POLE-mutant endometrial cancers and found that these cases were associated with a favorable prognosis (11). Moreover, POLE mutant ECs have a high tumor mutational burden (TMB) and increased lymphocyte infiltration, which is closely associated with immunotherapy response (12–15). Among them, P286R and V411L are the most common hotspot mutation sites in the POLE gene (16). In our previous study, we found that POLE mutations regulate glucose metabolism in the body and led to a good prognosis (17). In addition, POLE mutations were found to be significantly associated with responses to adjuvant therapy, and patients with POLE mutations receiving chemotherapy exhibited poorer overall survival (OS) (18), which may be due to the generation of many tumor neoantigens by POLE mutations, resulting in patient insensitivity to adjuvant therapy.

Genetic mutations lead to tumorigenesis, and certain nonsynonymous mutations can result in peptides that, after being degraded by the proteasome, may bind to major histocompatibility complexes (MHCs) and be presented to antigen-presenting cells (APCs). Short peptides with MHC binding motifs, named “neoantigens”, are recognized by T-cell surface factors to generate an immune response that triggers tumor regression. These antigens are endogenous, recognized by T cells as “foreign” and less susceptible to interference by immune tolerance mechanisms, thus becoming effective targets for tumor immunotherapy (19, 20). With the advent of next-generation sequencing (NGS) technologies, the exploration of tumoral neoantigens has emerged as a novel frontier in immunotherapy. These tumor-specific tumoral neoantigens are uniquely expressed in tumor cells, offering highly targeted therapeutic opportunities (21). Currently, a combination of tumor sequencing and p-MHC prediction allows the identification of candidate tumor neoantigen peptides in tumor patients, leading to the synthesis of personalized peptides for tumor-targeted therapy (22–26). Neoantigens are caused by mutations in the tumor genome, making them ideal candidates for targeting tumors. In recent years, significant progress has been made in peptide complex-targeted neoantigen therapies. By using computational biology methods, researchers can predict potential tumor neoantigens and design corresponding peptides. These peptides can bind to human leukocyte antigen (HLA) molecules to form peptide complexes, which are then recognized by tumor cell-specific T cells, triggering an immune response that ultimately leads to tumor cell lysis (27–29). The POLE gene is the largest catalytic subunit of human DNA polymerase with DNA polymerase activity and nucleic acid exonuclease correction activity, and it plays an important role in cellular DNA replication and base mismatch recognition and repair and is responsible for chromosomal DNA replication during cell division (30–32). Early studies showed that POLE mutations were observed in colorectal cancer, pancreatic cancer, ovarian cancer, high-grade glioma and EC (33–37). Among them, EC is the malignancy with the highest percentage of known POLE gene mutations, with mutation rates of 5-10% (38, 39).

The present study aimed to reveal the high immunogenicity of POLE-mutant EC. Neoantigens generated by the POLE^P286R^ mutation were identified by screening. The design of peptide-MHC complexes that target the neoantigens generated by the POLE^P286R^ mutation to kill EC tumor cells by specific T cells may be a new direction for immunotherapy of POLE^P286R^ EC.

## Results

### Characteristics of the immune microenvironment in EC patients with POLE mutations

**Figure 1:**
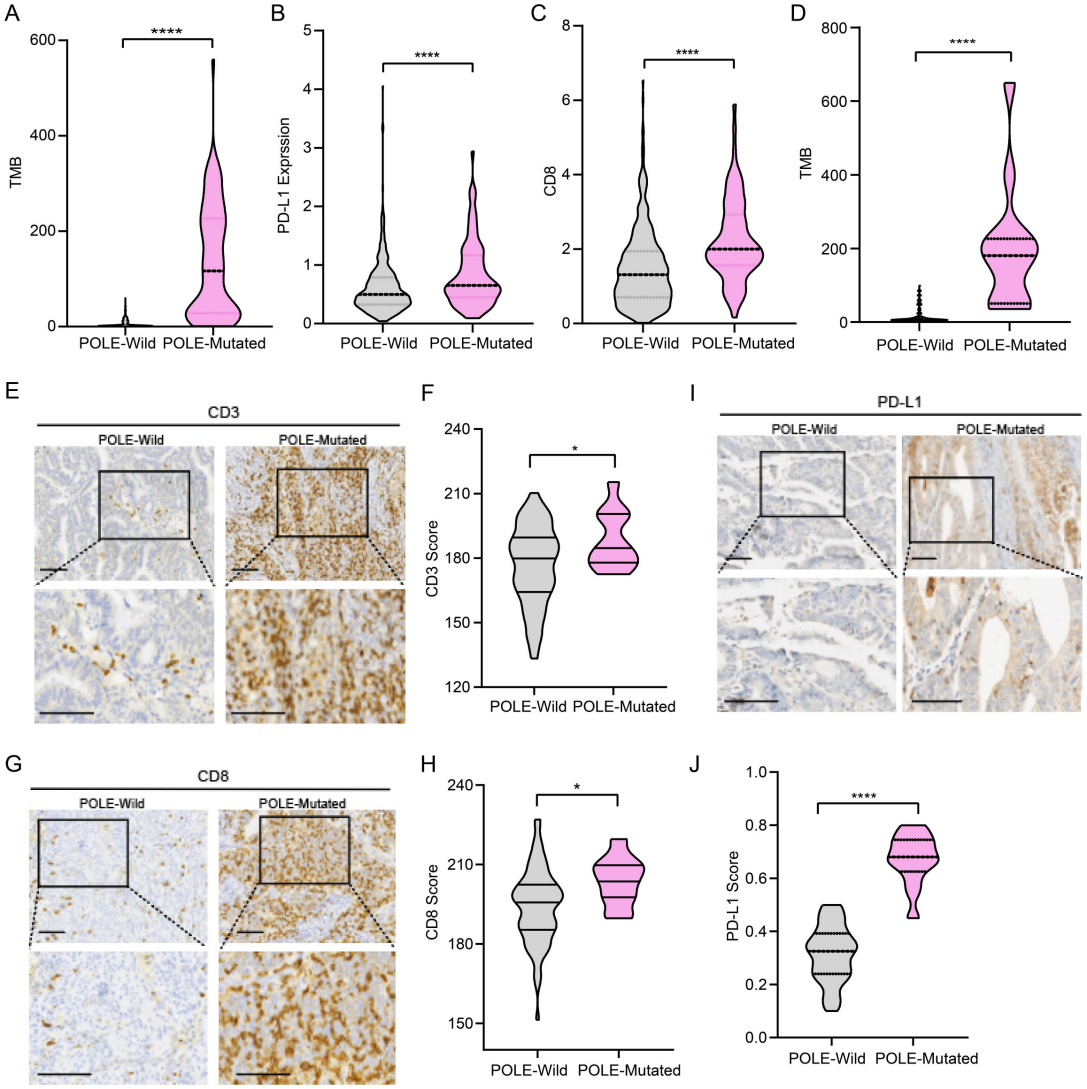
Immune microenvironment characteristics of POLE-mutant endometrial cancer (EC). **(A)** Comparison of tumor mutation burden (TMB) between POLE mutant versus wild-type EC patients, data from TCGA. **(B)** Comparison of PD-L1 expression between POLE mutant versus wild-type EC patients, data from TCGA. **(C)** Comparison of CD8^+^ T-cell infiltration between POLE mutant versus wild-type EC patients, data from TCGA. **(D)** Comparison of TMB between POLE-mutant and WT EC patients (data from 99 EC patients from Shanghai First Maternal and Child Health Hospital). **(E, F)** Representative images of immunohistochemical staining for CD3 in POLE control and POLE-mutant samples **(E)**. Scale bar=50μm. Comparison of the CD3 score of immunohistochemical staining between POLE mutant versus wild-type EC patients **(F)**. **(G, H)** Representative images of immunohistochemical staining for CD8 in POLE control and POLE-mutant samples **(G)**. Scale bar=50μm. Comparison of the CD8 score of immunohistochemical staining between POLE mutant versus wild-type EC patients **(H)**. **(I, J)** Representative images of immunohistochemical staining for PD-L1 in POLE control and POLE-mutant samples **(I)**. Scale bar=50μm. Quantification of PD-L1 immunohistochemical staining scores in POLE control and POLE-mutant samples **(J)**. Data from 99 EC patients from Shanghai First Maternal and Child Health Hospital in **(E-J)**. * p<0.05, ****p<0.0001.

To reveal the immunogenicity profile of POLE mutations in EC, publicly available data from the cBioPortal database (http://www.cbioportal.org/) were applied for the analysis. We observed higher TMB scores in patients with POLE mutations than in wild-type (WT) patients (**Figure 1A**) Additionally, CD8^+^ T lymphocyte infiltration and PD-L1 expression showed significant differences between WT and POLE-mutated patients (**Figures 1B, C**). To further validate the results, we analyzed 99 clinical samples of EC patients from Shanghai First Maternal and Child Health Hospital (40). The results also revealed that the TMB scores were higher in patients with POLE mutations than in WT patients (**Figure 1D**), suggesting that POLE mutations may lead to the production of neoantigens, which can trigger an immune response and potentially enhance the immunogenicity of the tumor. To further evaluate the relationships between POLE mutations and the immune microenvironment, immunohistochemical staining was performed to compare the numbers of CD3^+^, CD4^+^ and CD8^+^ T cells between WT and POLE-mutated patients from 99 clinical EC samples. The results revealed that the numbers of CD3^+^, CD4^+^ cells as well as CD8^+^ T cells were significantly greater in POLE-mutated patients than in WT patients, indicating the involvement of the immune microenvironment in POLE-mutated EC patients (**Figures 1E–H**, **Supplementary Figure S1**). In addition, immunohistochemical staining for PD-L1 showed that higher PD-L1 expression in patients with POLE mutations than in WT patients (**Figures 1I, J**), suggesting a potential mechanism of immune evasion by the tumor cells. Overall, these results contribute to our understanding of the EC immunogenicity profiles in patients with POLE mutations and support the notion of a more favorable immune microenvironment in POLE-mutant EC, which may have the potential for utilizing immunotherapy strategies targeting the immune microenvironment in these patients.

### Identification of high-frequency sites for POLE mutations and neoantigen prediction in patients with EC

**Figure 2:**
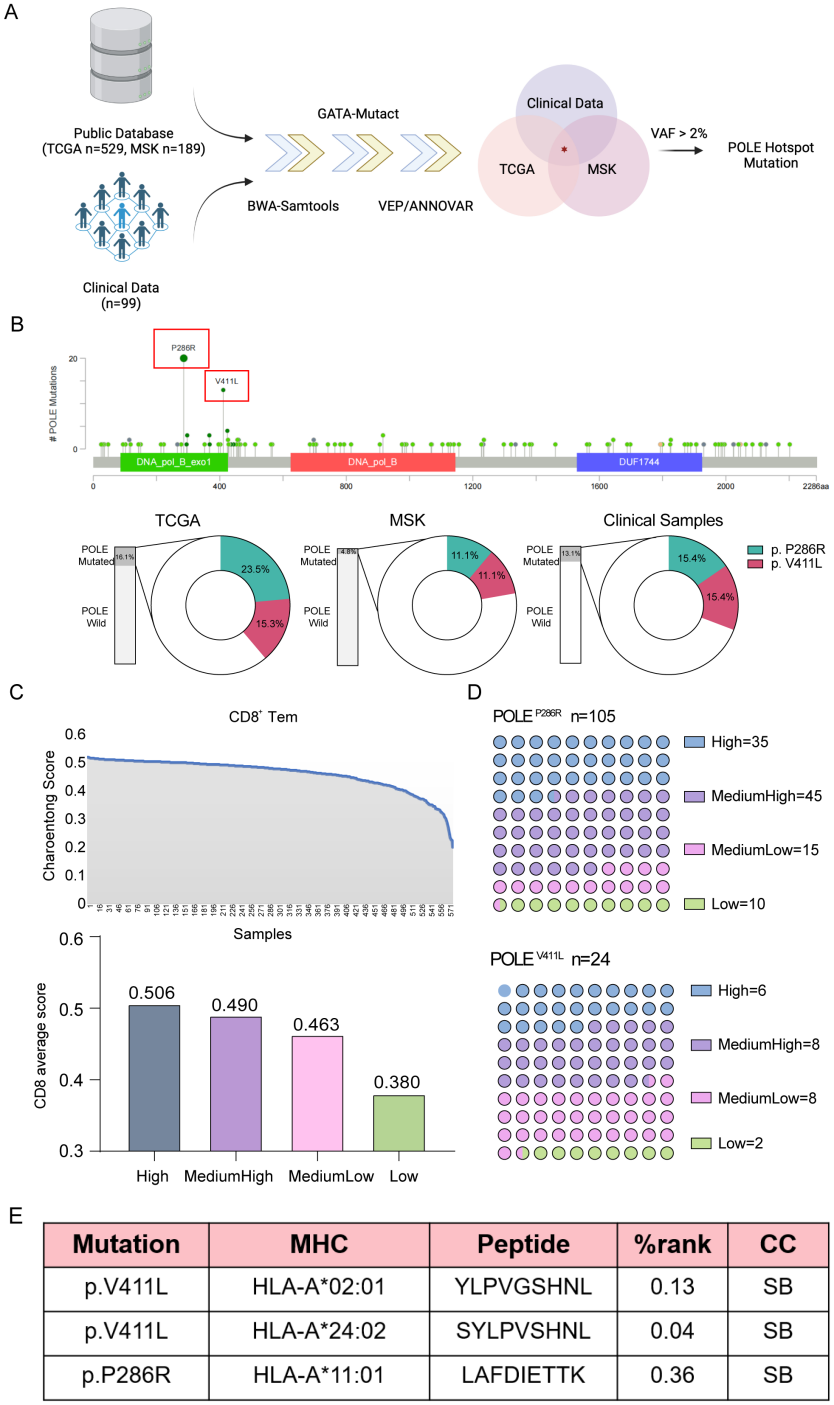
Analysis of tumor lymphocyte infiltration and the extent of lymphocyte infiltration at relevant mutated loci. **(A)** Screening process for POLE hotspot mutations in EC. Data from TCGA and MSK datasets and our clinical samples were analyzed. **(B)** Mutational sites in the POLE gene were identified using TCGA data (upper), and the proportions of POLE mutations in the TCGA and MSK datasets and our clinical samples were analyzed (lower). The proportions of two POLE hotspot mutations, namely, POLE^V411L^ and POLE^P286R^, were shown. **(C)** The CD8^+^ T-cell content of each specimen was calculated using Charoentong signatures in transcriptome sequencing data from TCGA and MSKCC for EC. The horizontal axis showed the samples, and the vertical axis showed the CD8^+^ T-cell content score (Charoentong score), ranked from the maximum to the minimum. Then, the 583 specimens were divided into four classes according to the CD8^+^ T-cell score and labeled high, medium-high, medium-low, and low content. **(D)** Analysis of the numbers of EC patients with different degrees of tumor lymphocyte infiltration at the associated POLE mutation locus. **(E)** Tumor neoplastic antigen prediction. The software predicted three high-frequency MHC class I molecules (HLA-A*02:01, HLA-A*11:01, HLA-A*24:02) in the Chinese population and determined the binding relationship between HLA and mutant antigen peptide chains. %rank<0.5, the short peptide is considered to bind strongly to MHC-I class molecules; 0.5<%rank<2, the short peptide binds weakly to MHC-I class molecules; %rank>2, the peptide cannot bind to MHC-I class molecules. If the predicted peptide binds more strongly to MHC class I molecules, it is more likely to be a neoantigenic epitope. CC, combining capacity; SB, strong binding.

To further identify antigens resulting from POLE mutations, we intersected the mutation information from public data sources (TCGA and MSK datasets) and 99 clinical EC samples, and obtained 91 shared mutants, among which two POLE hotspot mutations, POLE^P286R^ and POLE^V411L^, were identified as the two most frequent POLE mutations (**Supplementary Table S1**, **Figure 2A**). In the TCGA dataset, these two mutations had the highest proportions of mutations in the loci, namely POLE^P286R^ at 23.5% and POLE^V411L^ at 15.3%. These two mutations were also identified in the MSKCC database with POLE^P286R^ at 11.1% and POLE^V411L^ at 11.1%. Among the 99 EC clinical samples, 13 samples showed POLE mutations. Specifically, 2 samples (15.4%) each exhibited the POLE^P286R^ and POLE^V411L^ mutations (**Figure 2B**).

The transcriptome sequencing data, from 583 cases of endometrial cancer (EC) obtained from the TCGA and MSKCC databases, had corresponding exon sequencing mutation data available. The CD8^+^ T-cell content of each specimen was calculated by using Charoentong signatures, and the patients were distinguished as high, medium-high, medium-low, and low according to the CD8^+^ T-cell count score; the average score values for these groups were 0.506, 0.490, 0.463, and 0.380, respectively (**Figure 2C**). The number of patients with varying degrees of tumor lymphocyte infiltration at the relevant POLE gene mutation locus was also analyzed (**Figure 2D**). CD8^+^ T lymphocyte infiltration at the two high-frequency mutation sites was counted according to the above CD8^+^ T-cell score. The results suggested that the number of high- and intermediate-high-grade tumors was greater than the number of intermediate-low- and low-grade tumors, suggesting increased infiltration of POLE mutant CD8^+^ T lymphocytes. Since the focus of this study was on the cytotoxic killing effect of nascent antigen-activated CD8^+^ T cells, the antigen-presenting HLA molecules were mainly HLA class I molecules. Using NetMHCpan 4.1 software for prediction, the high-frequency MHC class I molecules in the Chinese population were found to be HLA-A*02:01, HLA-A*11:01, and HLA-A*24:02, and the POLE mutant antigen peptide sequences that combined with these high-frequency MHC class I molecules were screened. HLA-A*02:01 and HLA-A*24:02 bound stably to the POLE^V411L^ antigen peptide, while HLA-A*11:01 stably and strongly bound to the POLE^P286R^ antigen peptide. Finally, we identified two high-frequency sites of the POLE mutation and three new antigenic peptides resulting from this mutation (**Figure 2E**).

### The capture of specific T cells using synthesized POLE^P286R^ mutant antigens

**Figure 3:**
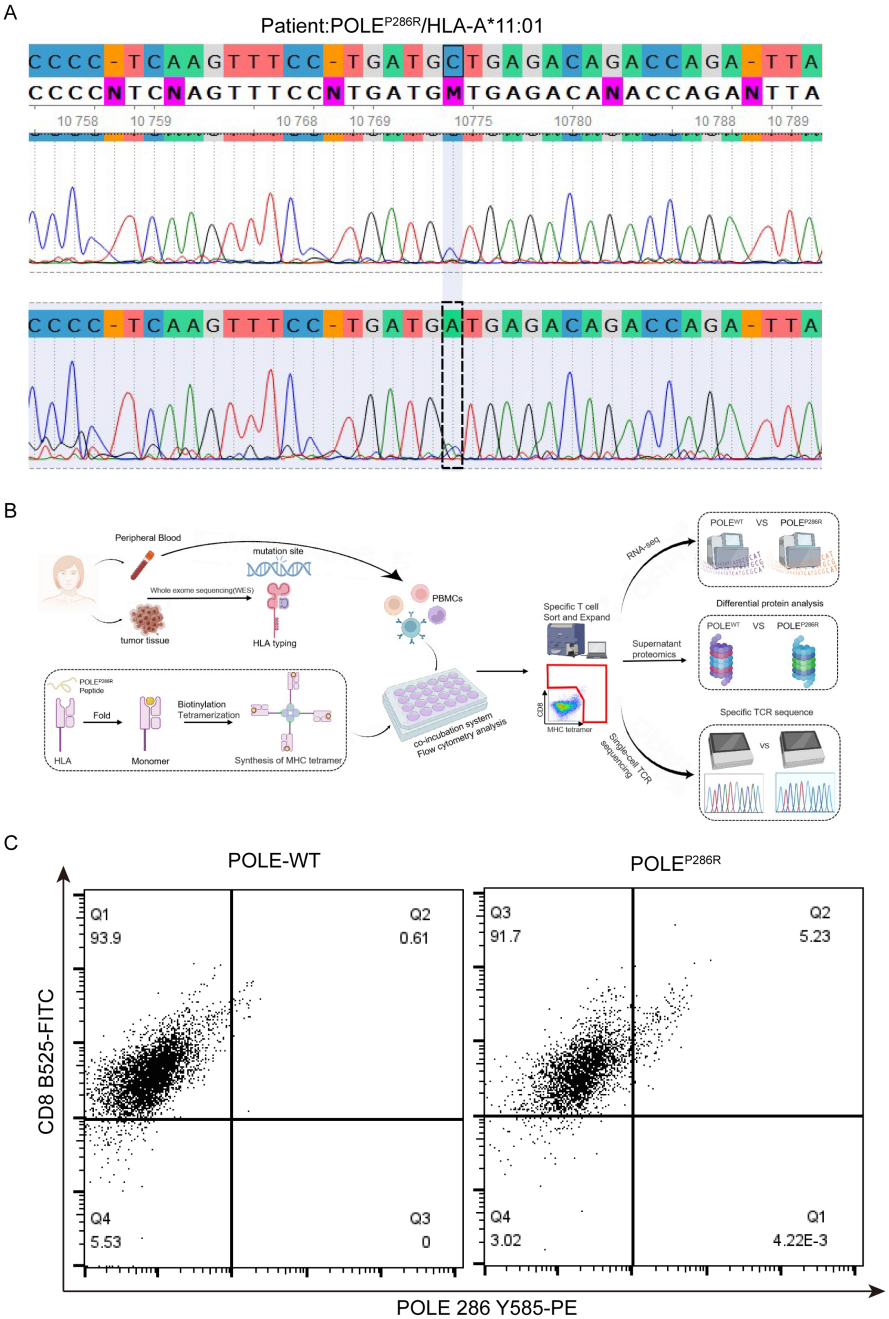
Synthesis of POLE mutant antigens and capture of specific T cells. **(A)** Second-generation sequencing confirmed the POLE mutation locus in this patient with EC. **(B)** Synthesis process of POLE^P286R^ mutant MHC complex tetramers and the capture process of specific T cells. **(C)** Flow cytometry captures double-positive specific T cells. Flow cytometry analysis of the POLE^P286R^ mutant tetramer with a CD8 antibody. Values in the upper right quadrant indicate the percentage (%) of POLE mutant antigen pMHC tetramer-positive cells in CD8^+^ cells from the peripheral blood of EC patients with the POLE^P286R^ mutation.

Based on the above identified POLE hotspot mutation locus, we searched for EC patients with POLE mutations in our clinical work. Among 76 EC patients, we found only one patient whose tumor tissue had the POLE^P286R^ mutation and a matched HLA-A*11:01 genotype (**Figure 3A**). Therefore, peripheral venous blood was collected from this patient, and PBMCs were isolated for the follow-up study. Moreover, we used the predicted POLE^P286R^ antigen peptide (LAFDIETTK) as shown in **Figure 2E**, to synthesize the epitope peptide. Subsequently, the antigen peptide fragment was added into the recombinant protein MHC class I heavy chain and β2 microglobulin (β2m) to be expressed in Escherichia coli (*E. coli*), and an MHC tetramer was constructed by peptide folding, monomer biotinylation and tetramerization, thus completing the preparation of the T-select MHC tetramer (**Figure 3B**). CD8^+^ T cells bound to the POLE^P286R^ peptide-MHC complex tetramer were detected in PBMCs, and POLE^P286R^ antigen-specific T cells were screened and amplified. The POLE^P286R^ mutant antigen peptide MHC-I complex was enriched in a high percentage of CD8^+^ T cells compared to controls (POLE^WT^). We identified the POLE^P286R^-HLA-A*11:01 tetramer-enriched CD8^+^ T cells by flow cytometry. The proportion of tetramer-positive cells in WT control was only 0.61%, while it was 5.23% in the POLE^P286R^ group (**Figure 3C**). This finding indicated the presence of POLE^P286R^ mutant antigen-specific T cells in EC patients with POLE mutations.

### Identification of the POLE^P286R^ mutant neoantigen-reactive TCR

**Figure 4:**
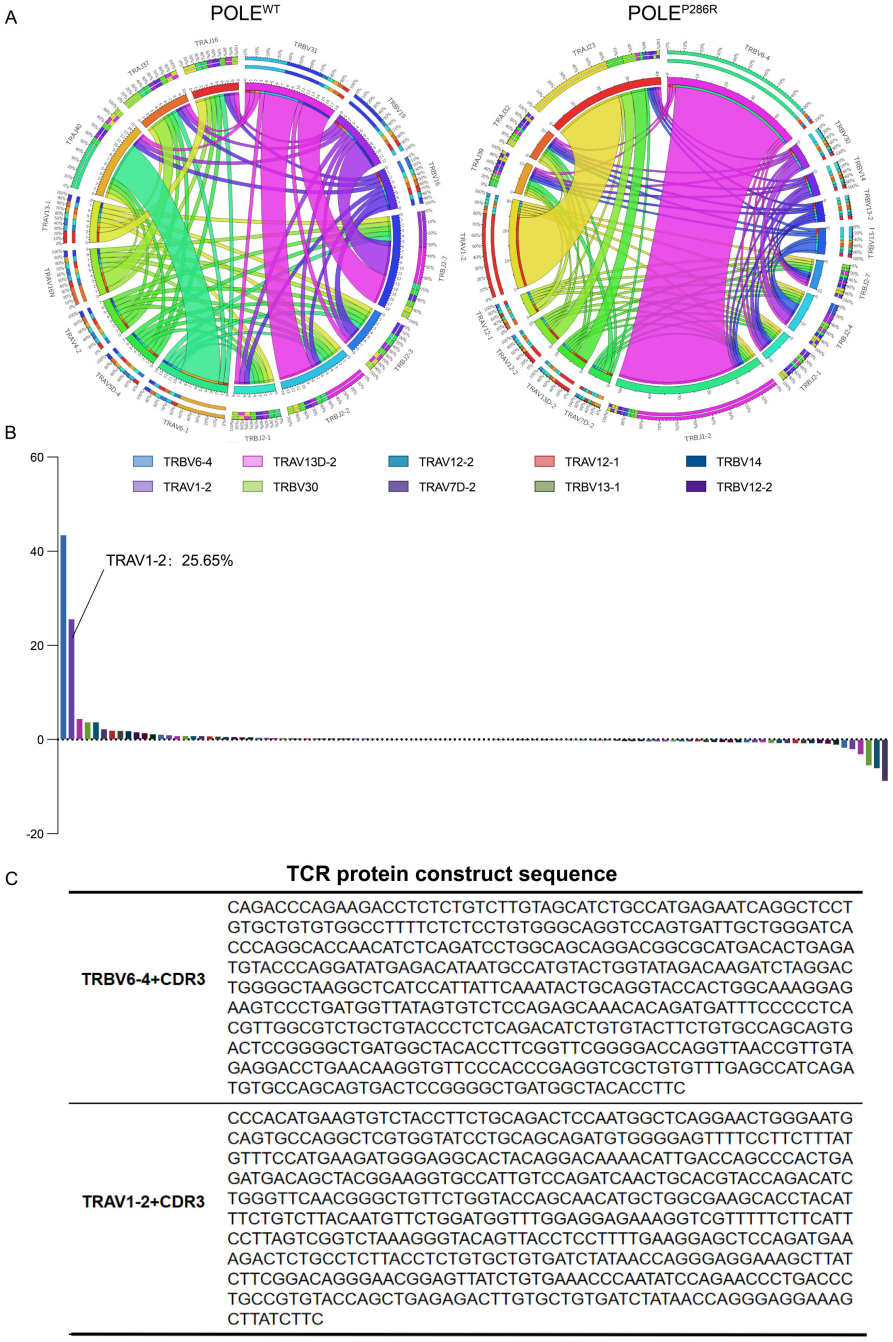
TCR sequencing was used to identify the specific TCR sequences for POLE^P286R^ neoantigens. **(A)** TCR structural rearrangement map. POLE^P286R^ mutant group with dominant rearrangement. **(B)** Frequency plot of the distribution of the difference clone table. The horizontal axis is the proportion of each TCR V region gene in the total TCR clones, and the vertical axis is the TCR V region gene type. The frequency plot of the distribution of the differential clone table shows that TRBV6-4 and TRAV1-2 constitute the specific clones of POLE^P286R^, accounting for 43.5 and 25.65% of all T cells, respectively. The symbols of 10 clones with the highest frequency are shown. **(C)** TCR protein sequences. TRBV6-4 and TRAV1-2, as well as their unique CDR3 region, constitute the main protein sequences for binding neoantigens.

CD8^+^ T cells from the peripheral blood of POLE^WT^ or POLE^P286R^ patients were collected separately and analyzed by TCR sequencing. TCR is formed by multiple combinations of V, D, and J genes. Each VDJ combination forms a unique and variable TCR structure, while the highly variable antigen-recognizing region on the V gene of the TCR is the CDR3 region (complementarity determining region, CDR), the amino acid sequence of which determines the type of antigen recognized. The combination of the VDJ and CDR3 regions in the TCR determines the uniqueness of the TCR clone. We compared the number of clones of each TCR VDJ combination and the sequence of the CDR3 region in the POLE^WT^ and POLE^P286R^ groups (**Figure 4A**). The width of the line between TRBV6-4/TRBJ1-2 and TRAV1-2/TRAJ23 in the TCR rearrangement map represents the proportion of all TCR types; the higher the proportion is, the greater the number of T cells with rearranged TCR clones. The TRBV6-4/TRBJ1-2 rearrangement (purple region) and the TRAV1-2/TRAJ23 rearrangement (yellow region) were the dominant rearrangements seen in the POLE^P286R^ group. The frequency plot of the distribution of the differential clone table shows that TRBV6-4 and TRAV1-2 constituted the specific POLE^P286R^ clones, accounting for 43.5 and 25.65% of all T cells, respectively (**Figure 4B**), while these clones were not present in POLE^WT^ group. TRBV6-4 and TRAV1-2, together with their specific CDR3 region, constituted the main protein sequence for binding the target antigen (**Figure 4C**). This led us to identify specific TCRs against the POLE^P286R^ neoantigenic peptide, which can be used for subsequent validation of the antitumor effect and provide new immunotherapeutic ideas for patients with POLE^P286R^ mutant EC.

### Expression profiles of CD8^+^ T cells specific for POLE^P286R^ mutant antigens

**Figure 5:**
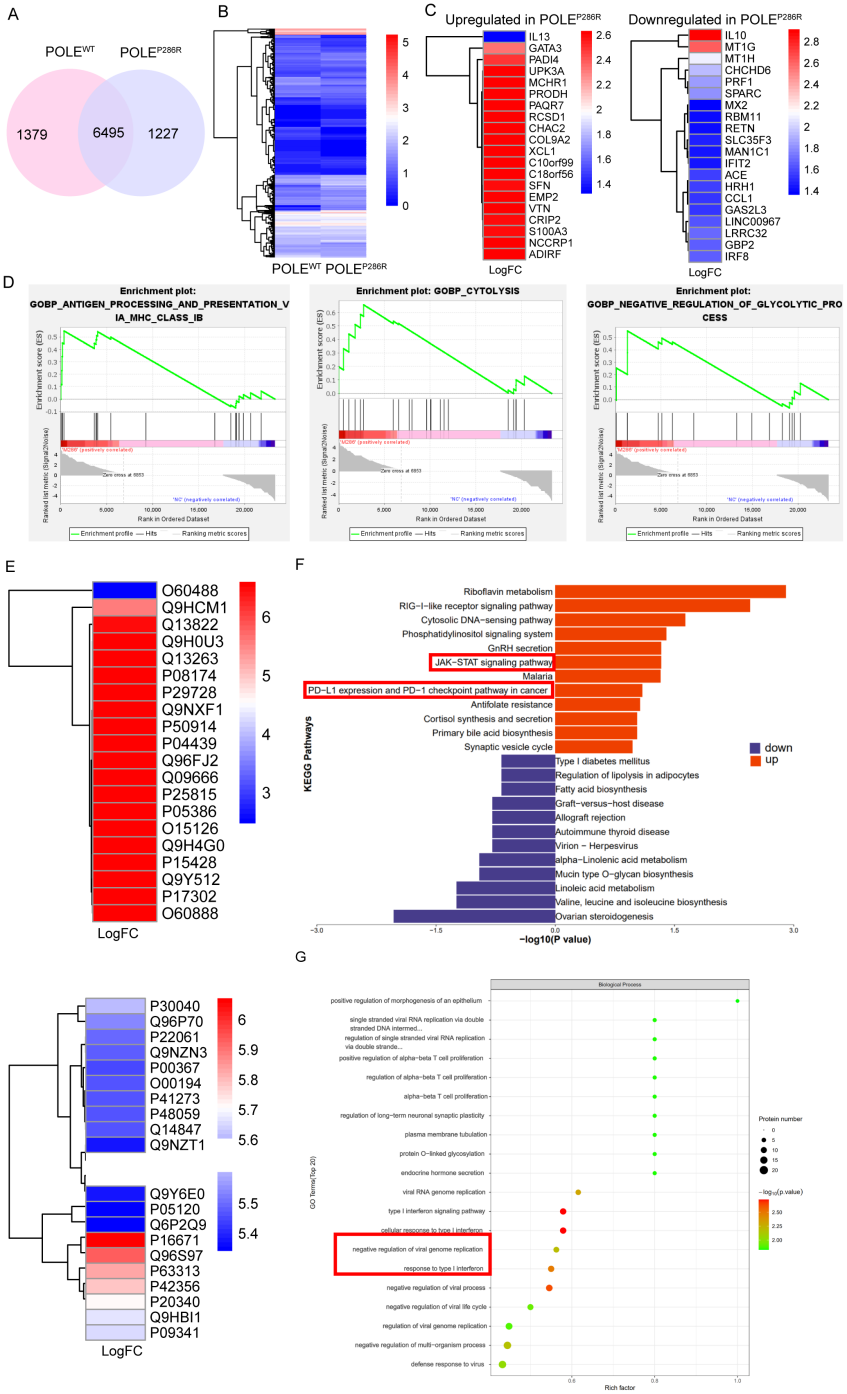
Gene expression and proteomic characteristics of T cells specific for POLE^P286R^ mutant antigens. **(A)** Venn diagram showing the high number of differentially expressed genes between the two sample groups. **(B)** Cluster analysis showed a clear distinction between the two groups of differentially expressed genes. **(C)** Screening of 20 genes with the most significantly high (left) and low (right) expression in the POLE^P286R^ mutant group compared to in the POLE^WT^ group. **(D)** GSEA indicated that POLE^P286R^ mutant antigen-specific T cells significantly activated multiple tumor- and immune-related signaling pathways. **(E)** T-cell cultures were subjected to proteomic analysis, and the differentially expressed proteins that were significantly up- and downregulated in the POLE^P286R^ group were listed separately. **(F)** KEGG analysis showed that POLE^P286R^ upregulated tumor- and immune-related signaling pathways. **(G)** GO enrichment analysis revealed active developmental and metabolic regulation in the POLE^P286R^ group of cells, with a functional focus on signaling pathways associated with the interferon response.

For further insight into the differences between the two types of T cells, POLE^P286R^ mutant antigen-specific T cells from POLE^P286R^ group and control T cells from POLE^WT^ group were used. The specific CD8^+^ T cells obtained in the previous step were cultured, amplified, and collected, and transcriptome sequencing was performed. More than 6000 differentially expressed genes (DEGs) were found between POLE^WT^ and POLE^P286R^ groups (**Figures 5A, B**). The top 20 genes with the most significant high and low expression in the POLE^P286R^ mutant group were screened separately (**Figure 5C**). It is important to recognize that IL-10 has complex roles in the tumor microenvironment. While it is associated with immunosuppression, particularly through inhibition of Th17 cells and modulation of macrophage activity, there is evidence suggesting that IL-10 can also contribute to anti-tumor immune responses under certain conditions (41, 42). In our study, the elevated IL-10 expression may reflect an adaptive immune response attempting to balance pro-inflammatory and anti-inflammatory signals in the presence of tumor antigens. Analysis of the functional differences between POLE mutant antigen-specific T cells and controls using the GSEA algorithm showed that POLE^P286R^-HLA-A*11:01-specific T cells significantly activated several tumor- and immune-related signaling pathways; the more significantly different pathways were those related to antigen presentation and processing, cytolysis and glycolysis (**Figure 5D**).

To further compare the functional differences between POLE^P286R^ and POLE^WT^ groups, we collected T-cell culture supernatants for proteomic analysis, and the proteins that were significantly up- and down-regulated in the POLE^P286R^ group are listed in **Figure 5E**. GO and KEGG enrichment analyses were performed. Consistent with the transcriptomic information, KEGG analysis of the differentially expressed proteins showed that in the POLE^P286R^ group, tumor- and immune-related signaling pathways, such as the JAK-STAT signaling pathway and the immune checkpoint PD1/PD-L1 signaling pathway, were upregulated (**Figure 5F**). These pathways are indeed interconnected and play pivotal roles in tumor immunity. The activation of JAK/STAT signaling is known to promote the production of cytokines such as IFN-γ, which can upregulate PD-L1 expression on tumor cells, thereby contributing to immune evasion (43). Our findings suggest that the POLEP286R mutation may drive T cell responses that inadvertently enhance PD-L1 expression, leading to a feedback loop that complicates anti-tumor efficacy. The result of GO enrichment analysis showed that the POLE^P286R^ group was active in cell development and metabolic regulation, with a functional focus on interferon response-related signaling pathways, which are closely related to the immune response (**Figure 5G**, **Supplementary Figure S2**).

Synergistic transcriptomic and proteomic analyses revealed that POLE^P286R^ mutation-specific T cells significantly activated tumor- and immune-related signaling pathways upon recognition of POLE neoantigens, further confirming the possibility of neoantigen production by mutations at this locus.

### EC organoids confirmed the antitumor effect of the POLE^P286R^ neoantigen and specific T cells

**Figure 6:**
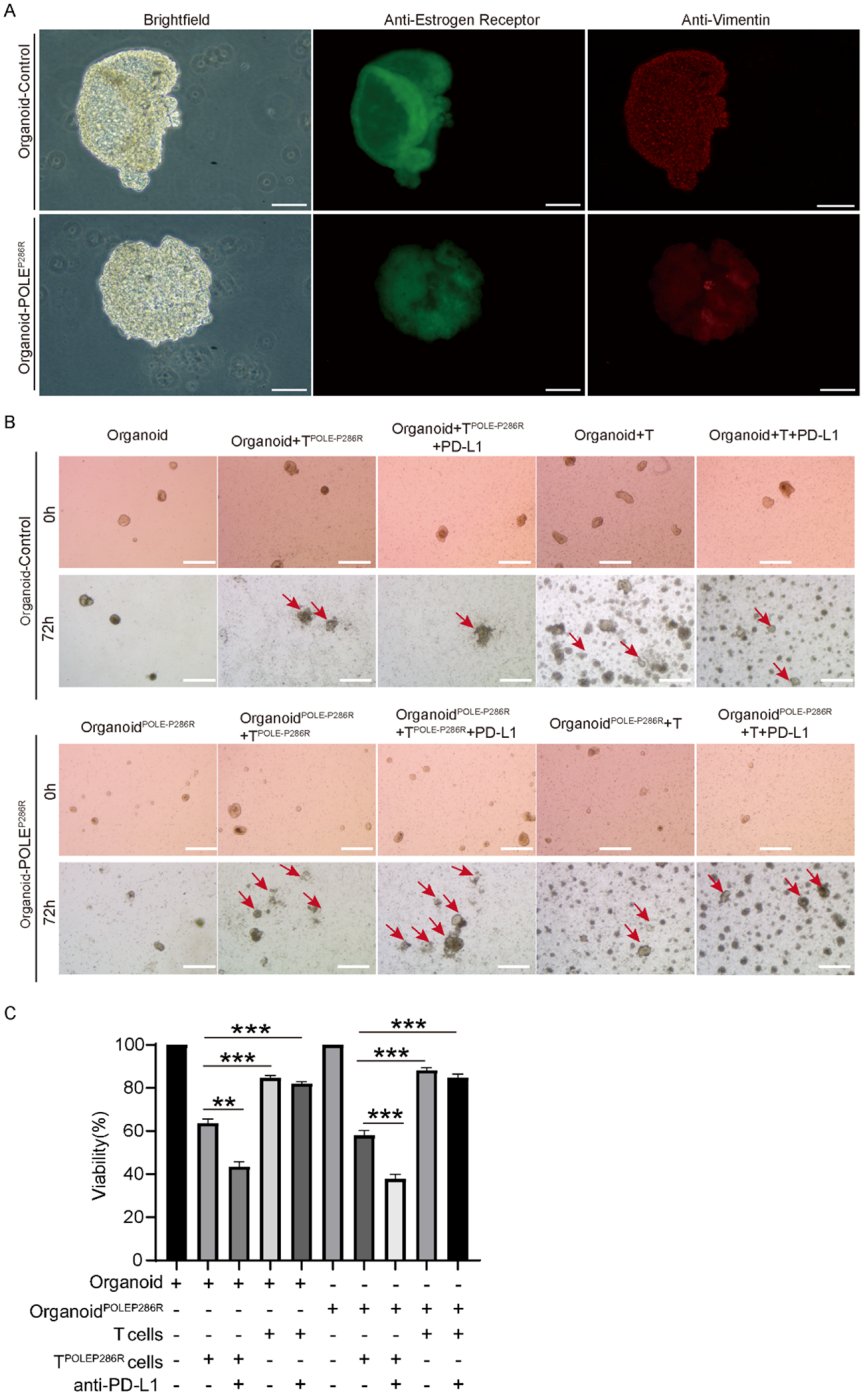
EC organoids confirmed the antitumor effect of the POLE^P286R^ neoantigen and specific T cells. **(A)** Immunofluorescence staining confirmed EC and an organoid, scale bar=100μm. Anti-Estrogen staining in green, and anti-Vimentin staining in red. **(B)** The organoid assay confirmed the antitumor effect of specific T cells and increased killing by the anti-PD-L1 antibody, scale bar=500μm. Red arrows indicate the killing effects. **(C)** T^POLE-P286R^ had a killing effect on organoid^POLE P286R^ and control organoid and the killing effect was enhanced by the addition of PD-L1. **p < 0.01, ***p < 0.001.

To further validate the tumor-killing effect of the neoantigen generated by the POLE^P286R^ mutation with specific T cells, we collected tissues and blood from EC patients, constructed organoid models, and cocultured them with isolated PBMCs. During the experiment, we collected postoperative endometrial tissues from two EC patients, one of whom had tumor tissues without the POLE mutation and served as a control group, and the other patient with the POLE^P286R^ mutation that we mentioned before was used as an experimental group for organoid culture. Immunofluorescence (IF) staining confirmed that the organoids were in good condition (**Figure 6A**). We cocultured the two groups of organoids with specific T cells (peripheral blood T cells from patients with the POLE^P286R^ mutation) and nonspecific T cells (peripheral blood T cells from patients without the POLE mutation), respectively. The experimental results confirmed that T cells (POLE^P286R^) had a killing effect on organoid^POLE-P286R^ and nonmutated organoids, with the killing effect being more pronounced in the experimental group. Nonspecific T cells had no killing effect on either group of organoids. The experimental results fully demonstrated that the POLE mutation at this locus produced a new antigenic peptide, and the T cells generated against this antigen were found to have specific tumor-killing effects (**Figures 6B, C**). Based on the results of previous studies, we further speculate that patients with POLE mutations are more sensitive to immunotherapy, so we further added anti-PD-L1 antibody (Adebrelimab Injection, Hengrui Medicine Co., Ltd., S20233106) to the experimental group to determine whether the treatment combined with anti-PD-L1 antibody further promoted the killing of tumors. The final experimental results confirmed a more prominent killing effect in the mutant group with the addition of the PD-L1 antibody. This finding suggests that people with this POLE mutation site may benefit more from immunotherapy, leading to an improved prognosis. This finding will provide new individualized therapeutic strategies for the endometrial cancer population with the POLE^P286R^ mutation.

## Discussion

This study focused on the relationship between POLE mutations and the immune microenvironment of EC, a common malignancy in women. According to our previous studies, POLE mutant EC is characterized by a hypermutated phenotype, increased tumor mutation burden, and increased lymphocyte infiltration, all of which are closely associated with the immune response and may lead to more benefits from immunotherapy, resulting in improved disease prognosis. Patients with POLE mutations have also been described in the literature to have a significant response to immune checkpoint inhibition, and cancers harboring POLE mutations are good candidates for treatment with immune checkpoint inhibitors, increasing the likelihood that conservative treatment will be available for younger patients. In patients with multiple metastases, metastatic tumors shrink significantly and treatment outcomes are improved (44–47).

Given that tumor neoantigens are a new direction for EC immunotherapy, we explored and characterized neoantigens produced by EC with the POLE^P286R^ mutation. We obtained results consistent with previous studies through statistical analysis of information from clinical samples and public databases, fully demonstrating that the POLE gene plays an immunogenic role in EC. While both POLE^P286R^ and POLE^V411L^ mutations are the high prevalent mutations in our study, we were only able to collect samples with the POLE^P286R^ mutation for detailed experimental investigation, which is mainly due to the limited numbers of the available clinical specimens that met our study criteria. Therefore, we cannot definitively conclude that POLE^V411L^ exhibits similar properties to POLE^P286R^ without further studies. Further experiments will be necessary to elucidate the functions of the POLE^V411L^ mutation in EC. Therefore, we focused on POLE^P286R^ in the present study. We found that the altered immune microenvironment was caused by a POLE mutant antigen and identified a POLE^P286R^ mutant neoantigen and the corresponding MHC complexes (HLA-A*11-01p.P286R).

In 2015, a study by Howitt et al. showed that POLE mutant EC produced 15 times more tumor neoantigens than MSI EC and more than 100 times more neoantigens than MSS EC (48). The Phase Ib clinical trial KEYNOTE-028 reported that a POLE-mutant advanced EC patient achieved partial remission and a sustained response for more than 14 months after 8 weeks of treatment with pembrolizumab (49). On the basis of the above studies, we further confirmed that POLE mutation EC is a tumor subtype with a high tumor mutational burden and good immunogenicity for molecular typing, which may be more effective for immunotherapy, by analyzing the database data and clinical sample data and combining them with immunohistochemical staining scores.

Previous studies have targeted RAS hotspot mutations, identified neoantigens and corresponding TCRs, and successfully validated the antitumor effects *in vivo* after TCR reconstruction and transduction (50). Neoantigens and specific TCRs, which were identified by FGFR3 mutations, also show unique antitumor toxicity (51). We believe that immunotherapies targeting shared neoantigens would constitute good approaches for cancer treatment. In our study, we successfully constructed MHC tetrameric complexes and captured POLE^P286R^ mutant antigen-specific T cells. Transcriptome sequencing and proteomics revealed that POLE mutant-specific T cells significantly activated tumor and immune-related signaling pathways upon recognition of the POLE antigen. In addition, through sequencing technology, we found that the antigen recognition receptor, TCR, of POLE^P286R^ mutant antigen-specific T cells had a significant oligoclonal distribution, demonstrating that the immunogenicity of POLE^P286R^ mutant antigen in patients stimulates the clonal proliferation of their recognition T cells. Eventually, the organoid model further validated the antitumor effects of specific T cells, suggesting the presence of neoantigens. The identification of peptides that are candidate EC neoantigens, the synthesis of personalized peptides, and the establishment of specific T-cell applications have made precision tumor immunotherapy modalities possible. Based on the above studies, there is a good reason to believe that the POLE^P286R^ mutation results in *the HLA-A*11:01 presentation of the mutant region peptide, which results in the formation of the pMHC complex on the surface of tumor cells. The complex is recognized by specific T cells to produce a killing effect.

Overall, based on the above studies, we propose the bold concept that POLE mutant peptides in EC can be used as immunotherapeutic targets to activate broad T-cell killing effects. We plan to establish cellular and animal models in subsequent studies to further identify POLE mutant EC neoantigen peptides, synthesize personalized peptides, and establish specific T-cell antitumor immunotherapy to provide molecular targets for guiding clinical precision therapy in the hope of improving the quality of survival and survival rate of POLE mutant EC patients.

In addition, it is worth mentioning that this study has several limitations, while some difficulties were encountered during the study. This major limitation may lead to a low generalizability of the research results. For the issue of sample size, we collected a total of more than 100 patients with EC during our preliminary case collection, and there was only one patient who had both the P286R mutation locus and HLA typing after performing tissue sequencing. While this one POLE^P286R^ organoid model demonstrated significant anti-tumor potential of the specific T cells, further validation using more patients-derived samples would strengthen our conclusions. We will actively search for cases with the same conditions for further validation in subsequent studies. We also need to pay attention to the uniqueness of the Chinese population. The specificity of the TCR clonal profile lacks stability during sequencing of T-cell receptors specific for POLE mutant antigens. To address this issue, we will continue to expand the sample size of eligible experiments for validation. Second, although we identified the sequence of the TCR protein that binds to this neoantigen, we have not validated the antitumor killing *in vitro* and *in vivo* at this time, which will be the focus of our subsequent studies. Despite these limitations, this is an important breakthrough for immunotherapy of this subtype of EC, making it possible to establish specific T-cell applications for tumor precision immunotherapy modalities. Previous studies have found that PD-L1 positivity can be used as a predictor of the prognostic status of EC patients. The increased involvement of PD-L1 in this study will also provide new evidence for the potential use of immunotherapy with neoantigens combined with PD-L1 in EC (52).

In brief, we found that POLE mutations were immunogenic in EC, confirmed the presence of the nascent antigen due to a high-frequency POLE mutation in EC, and obtained specific T cells against the POLE^P286R^ antigen, which have a specific antitumor effect. In addition, we identified the neoantigen recognition receptor (TCR) via sequencing technology. Importantly, these results suggest that the neoantigen peptide and TCR identified in this study are promising candidates for immunotherapy of POLE mutant EC. Due to the widespread presence of POLE mutations across cancers, such approaches could also be further explored and validated in other cancer diseases. Targeting the neoantigens generated by POLE mutations to obtain tumor-specific T cells is expected to be used in immunotherapy for various malignancies.

## Materials and methods

### Research data sources

Data source for clinical EC patients: exon sequencing data from 99 female EC patients from Shanghai First Maternal and Child Health Hospital (accession no: OEP001202. All data can be viewed in NODE: http://www.biosino.org/node) were applied for analysis of mutant amino acid types and human leukocyte antigen (HLA) types. Each patient dataset contained exome sequencing data from paired tumor tissue and peripheral blood samples. Public data were obtained from the cBioPortal database (http://www.cbioportal.org/), with the TCGA database including 529 cases and the MSKCC database including 189 cases of EC.

### Sequencing data analysis

#### Analysis of exon data

All public data specimens were compared using BWA-SAMtools analysis, while mutations were later identified using GATA-Mutect (GATK4.0) and annotated using VEP/ANNOVAR. The mutation information of 727 specimens from public data was intersected with that of the measured patient specimens, and the intersection strategy was as follows: the intersecting portion was identified by correlating the data from 3 sources using mutated gene names and pHGVS; the original intersection table in the result table (user data+TCGA+MSKCC data intersection) was based on the original data after correlation of the three data; and the data were filtered by applying the criterion VAF>2%. VAF represents the frequency of mutated loci in tumor tissues of patients and is a measure of mutational load.

Mutation loci that were common after mutation annotation as described above were filtered out. Then, we used the mutation transcript numbers and amino acid sequence numbers from the mutation annotation to obtain the complete results of exon mutant amino acid sequences using the hg38 genome sequence version from the RefSeq Protein sequence library (NCBI GenBank database) as the standard sequence. To predict the *de novo* antigenic peptide sequence of 8-15 amino acids, we intercepted 15 amino acids upstream and downstream of the mutation position to form the amino acid sequence to be predicted. The strategy used for mutation site selection was to select all missense mutations, frameshift mutations, and indel mutations in the coding region and to discard truncation mutations, nonsense mutations, and synonymous mutations.

#### Analysis of tumor lymphocyte infiltration

Tumor lymphocyte infiltration analysis was performed only for transcriptome sequencing data of EC from TCGA and MSKCC with 583 cases. All transcriptome sequencing specimens mentioned above had corresponding exon sequencing mutation data. The normalized postsequencing data from transcriptome sequencing were provided by the public data platform. The expression values for each gene used for analysis were normalized to gene length, thus reducing the effect of gene length on expression levels. Each gene expression level was then converted to a TPM value, which was defined as the percentage of each gene expression level (normalized by length) to the total sequencing volume per specimen. The CD8^+^ T-cell content was calculated for each specimen using Charoentong signatures (53), and the Xcell program was used as the calculation platform.

#### Analysis of the degree of lymphocytic infiltration of tumors at high-frequency POLE mutant loci

After Charoentong signatures were used to calculate the CD8^+^ T-cell content of each specimen, the 583 specimens were divided into four quartiles, labeled high, medium-high, medium-low, and low content, in order of CD8^+^ T-cell content from highest to lowest. The CD8^+^ T-cell content of each specimen corresponded to the mutation site and VAF value of that specimen.

### Immunohistochemistry

Paraffin-embedded tissue sections (4 μm) of EC specimens were subjected to immunohistochemical processing. First, specimens were dewaxed and dehydrated, followed by treatment with the anti-PD-L1 antibody (1:200; Cell Signaling Technology, 13684), anti-CD3^+^ antibody (1:200; Cell Signaling Technology, 85061), anti-CD4^+^ antibody (1:250; Abcam, ab133616) and anti-CD8^+^ antibody (1:500; Abcam, ab217344). After washing, the sections were incubated with biotin-conjugated secondary antibodies, followed by incubation with streptavidin-HRP. Sections were visualized by incubation with 3,3′-diaminobenzidine substrate (PR30010, Proteintech) and counterstained with hematoxylin. Images were acquired using a Mantra system (PerkinElmer, Waltham, Massachusetts, United States) with the same exposure time. The integral optical density was used to quantify the protein levels of PD-L1, CD3^+^, and CD8^+^ in the tumor tissue, and this integral optical density was calculated by dividing the staining intensity by the staining area (brown staining area).

### HLA typing and neoantigen candidate identification

Three high-frequency MHC class I molecules (54) (HLA-A*02:01, HLA-A*11:01, and HLA-A*24:02) were predicted in the Chinese population via the NetMHCpan4.1 software (55), and the binding relationship between the HLA antigen and POLE mutant antigen peptide chain was predicted. The detailed information was shown in **Supplementary Table S2**.

### Human subjects

Fresh human endometrial cancer specimens were collected from two endometrial cancer patients (one patient with WT POLE and the other patient with the P286R POLE mutation and HLA-A*11:01) after surgical tumor resection at Shanghai First Maternity and Infant Hospital. None of the patients received chemotherapy or immunosuppressive therapy at least 3 months prior to surgery. The use of tumor tissue samples for research purposes was in accordance with the guidelines of the Institutional Human Research Ethics Committee at the Shanghai First Maternity and Infant Hospital, Shanghai, China. Biopsies were obtained from surgical specimens measuring at least 2 x 2 x 2 mm in size. Collected tissue was transported in a solution of RPMI-1640 medium (Gibco, cat. no. 11875093), 1% penicillin/streptomycin, and 10 mM HEPES (Gibco, cat. no. 15630-056). If tissue was unable to be delivered on the day of the surgery, it was stored overnight at 4°C.

### Immunofluorescence staining

The organoid culture was transferred into a 1.5 ml centrifuge tube and briefly centrifuged. The supernatant was discarded, PBS was added to wash, the sample was further centrifuged and the supernatant was discarded. Then, 1 ml of 4% PFA was added, and the sample was fixed for more than 30 min at room temperature. The membrane was washed with PBS again, permeabilized with 0.1% Triton X-100 for 10 min. A diluted primary antibody (sc-6260, 1:100, Santa Cruz; ab32063, 1:200, Abcam) was added, and the membrane was incubated at 4°C overnight. The next day, the supernatant was centrifuged and discarded, diluted secondary antibody (A0423, 1:400, Beyotime) was added after PBS incubation for 3 hours at room temperature, and the supernatant was further centrifuged and discarded. The precipitate was aspirated, added dropwise to the slide, covered with a coverslip, and finally observed and photographed under a fluorescence microscope (BX53, Olympus).

### Tissue DNA extraction and mutation detection

After DNA extraction from frozen tissue samples using the AllPrep DNA/RNA/miRNA Universal Kit (Qiagen), agarose gel electrophoresis was performed to check for no degradation, and DNA samples were prepared as templates for PCR mutation detection. The primers for the POLE^P286R^ mutation and HLA-A*11:01 were shown in **Supplementary Table S3**. PCR mutation detection was performed using Taq DNA Polymerase Mix (Novozymes).

### MHC tetramer construction

Quantitative detection of antigen-specific CTLs was performed using MHC class I/peptide complex tetramers as probes (56). In the first step, the POLE^P286R^ mutant epitope peptide was synthesized at 95% purity (Genescript, Nanjing, China). Then, the recombinant proteins HLA-A*11:01 (Genescript, Shanghai, China) and β2 microglobulin (β2m) were expressed in *Escherichia coli*. After folding by adding the synthetic mutant peptide, the MHC class I/peptide complex monomer was formed and concentrated by ultrafiltration, and the folded complex monomer was obtained by molecular exclusion chromatography using an ÄKTA avant chromatography system (Cytiva). The A280 absorption peak was combined after chromatography using a HiLoad Superdex 75 pg (Cytiva). The fractions were collected in tubes. The lysine residue at the C-terminus of the MHC class I heavy chain in the complex monomer was biotinylated using the BirA enzyme, and the biotinylated complex monomer was again purified using the ÄKTA avant chromatography system (Cytiva) with HiLoad Superdex 75 pg (Cytiva) to obtain a single absorbance peak pure product. After molecular exclusion chromatography, the fractions that exhibited A280 peaks were combined. Finally, the purified biotinylated monomers were combined with streptavidin (Bioleng, Shanghai, China) labeled with the PE fluorochrome to form a tetramer, thus completing the preparation of the MHC tetramer.

### PBMC isolation, antigenic peptide stimulation and specific T-cell identification

Peripheral blood mononuclear cells (PBMCs) were prepared by taking 4 ml of peripheral blood from the patient with POLE^WT^ and the other patient with POLE^P286R^ mutation and matched HLA-A*11:01. Patient venous blood (4 ml) was collected in tubes containing ethylenediaminetetraacetic acid (EDTA; BD Biosciences, Oxford, UK). PBMCs were isolated by density gradient centrifugation with Ficoll-Paque (Histopaque; Sigma–Aldrich, Cambridge, UK). A total of 1x10^8^ PBMCs were obtained after isolating from peripheral blood. PBMCs were cultured in RPMI 1640 medium containing 10% FCS and 1% penicillin–streptomycin under normoxia (21% oxygen) and incubated overnight at 37°C in a 5% CO_2_ incubator. After overnight incubation, the suspended cells were plated on separate culture dishes and cultured. After addition of 2 ng of IL-4 (Sigma), 10 ng of the POLE^P286R^ mutant peptide and 2 ng of GM-CSF (Sigma) to the plated cells, cultivation was continued for 1 day, and 10 ng of the POLE^P286R^ mutant peptide was added every other day 5 times. Three days later, the T cells from the suspension culture were added and incubated with either *IL2* (10 ng/mL, Housheng Medical, Shanghai, China) or the PLOE^P286R^ mutant epitope peptide (synthesized by Genescript, Nanjing, China). At 21 days, all cells were collected and centrifuged at 2400 rpm for 20 min at room temperature and then resuspended in serum-free RPMI 1640 at a concentration of 1x10^6^ cells/ml. A total of 400 µl of cell suspension was taken, and PLOE^P286R^ –HLA-A*11:01 tetramer (5 µg/ml) was incubated for 30 min (maximum 4 h) at room temperature and protected from light.

After coincubation of POLE^P286R^ antigenic peptide-stimulated T cells with the tetramer, anti-CD8^+^ antibody (1:200; Cell Signaling Technology, 85336) was added, and the cells were incubated for 20 min at 4°C. The cells were resuspended by adding 100 µl of 10x PBS and centrifuged at 400 x g for 5 min at room temperature. After careful removal of the supernatant, the cells were resuspended in 200 μL of 0.1% BSA in PBS, and the number of antigenic peptide-specific T cells was detected by the CytoFLEX flow analysis platform.

### Transcriptome sequencing

RNA was extracted using a modified protocol for the RNeasy MinElute Clean-up Kit (Qiagen, Germany). Total RNA reverse transcription was performed via template conversion. Preparation of the reverse transcription system and execution of reverse transcription were then performed. As the second step, the mRNA library (reagents list: Vazyme #NR601; Vazyme #N411; Vazyme #N412; VAHTSTM RNA Adapters set 1-set 2 for Illumina) was enriched, the amplification system was prepared, the amplification procedure was performed, and Qubit quantification was performed. As the third step, a 50 ng sample was obtained based on the concentration measured in the previous step, prepared for fragmentation, and ligated in a PCR thermocycler. Finally, the library amplification procedure was executed, Qubit quantification was performed, and the library size distribution and concentration size were determined by agarose gel electrophoresis (nucleic acid analyzer). Sequencing was performed on the Illumina platform.

### 4D-label-free quantitative proteomics

PBMCs from the POLE^WT^ patient and the POLE^P286R^ mutant patient were isolated and cultured, respectively. For comparison of the differential changes in protein expression in the control and mutant groups, 4D-label-free quantitative proteomic techniques were used as reported previously (57). Cell culture supernatants of both groups were collected separately, and proteins were extracted by lysis (with 4% w/v SDT SDS, 100 mM Tris/HCl (pH 7.6), and 0.1 M DTT), followed by protein quantification by the BCA method. An appropriate amount of protein from each sample was subjected to trypsin digestion by the filter-aided proteome preparation (FASP) method (58). Then, the peptides were desalted, lyophilized and added to 40 μl of 0.1% formic acid solution for resolubilization, and the peptides were quantified (OD_280_). Each sample was separated using Easy nLC, an HPLC liquid phase system with a nanoliter-scale flow rate. The samples were separated by chromatography and analyzed by mass spectrometry using a timsTOF Pro mass spectrometer.

Mass spectral data were retrieved by MaxQuant (V1.6.6) software (Monitor Helix, China). Proteins with expression fold changes >1.2 (or <0.8) and P<0.05 were considered differentially expressed proteins (DEPs). DEPs were GO annotated using Blast2GO, and the KEGG database (http://www.kegg.jp/) was used to obtain information on pathway enrichment. GO and KEGG pathway enrichment analyses were performed using Fisher’s exact probability test.

### TCR sequencing

Total RNA was extracted from peptide-HLA-tetramer-positive cells. cDNAs were synthesized with a common 5’-RACE adapter from the total RNA using a SMART library construction kit (Clontech, Mountain View, CA, USA). By this method, T-cell receptor (TCR) α and TCRβ cDNA were amplified by PCR using the forward primer of the SMART articulator and the corresponding reverse primer (TCR primers purchased from Takara). After adding the Illumina index sequences with a barcode using the Nextera XT Index Kit (Illumina, San Diego, CA, USA), the prepared libraries were sequenced by 300-bp paired-end sequencing on the Illumina MiSeq platform using a MiSeq Reagent v3 600-cycles kit (Illumina, San Diego, CA, USA). The obtained sequence reads were analyzed using MiXCR (v3.0.3) (Bolotin DA [2015]) (59).

### Organoid culture, coculture and high-content imaging

Endometrial tumor tissue was collected, washed twice with PBS, and then minced in a Petri dish with shears into fragments of 1-3 mm^3^ or smaller. The fragments were transferred to a 15 mL Falcon tube with 5 mL of tumor tissue digestion solution. Then, tissue fragments were incubated in the solution at 37°C for 15-20 minutes and intermittently mixed every 5 minutes. Digestion was evaluated with light microscopy for dissociated fragments. Once at least 80% single cells were present in the mixture, PBS was added and filtered using a 100 μm cell strainer. The supernatant was transferred to a separate 15 mL Falcon tube and centrifuged at 300 x g for 5 minutes at 4°C. Next, the pellet was resuspended evenly in a 70:30 mixture of Matrigel^®^ Matrix (Corning, Catalog 356231). Organoid media were placed on ice to prevent polymerization of the mixture prior to plating. The mixture was plated into 50 µL domes on a prewarmed 24-well plate (Corning, Catalog 3524). The plate was placed in a CO2 incubator (37°C, 5% CO2) for 10 minutes to allow the Matrigel to polymerize and solidify, after which 0.75 mL of endometrial cancer organoid culture medium (JFKR, Catalog JFKR-EOC-100, Shanghai, China) was added to tumor endometrial organoid wells. The culture medium was changed every 2–3 days, and after 10 days of culture, the organoids were harvested for further processing.

Human endometrial cancer organoid culture medium (JFKR, Catalog FKR-EOC-100) contained Advanced DMEM-F12 (Gibco, cat. 12634-028), 50 ng/L Wnt3A (PeproTech, 315-20-2UG), 1% Glutamax (Life Technologies, Catalog 35050061), 1% HEPES ((Gibco, cat. 15630-056), 1% penicillin–streptomycin (Gibco, cat. 15070063), 2% B27 (Gibco, cat. 17504-044), 1% N2 (Gibco, cat. 17502048), 1% insulin-transferrin-selenium (Gibco, cat. 41400045), 0.2% Primocin (Gibco, cat. PML-40-60), 50 ng/mL human EGF (Peprotech, cat. AF-100-15), 100 ng/mL human FGF10 (Peprotech, cat. 100-26), 1.25 mM N-acetyl-L-cysteine (Sigma–Aldrich, cat. A9165-5G), 1 mM nicotinamide (Sigma–Aldrich, cat. N0636), and 0.5 mM A83-01 (Tocris, cat. 2939).

PBMCs from endometrial cancer patients were cultured in T-cell expansion basal medium (Gibco, cat. A1048503) with 100 U/ml IL-2 (PeproTech, cat. 200-02-50UG) and 3 µg/ml anti-human CD3/CD28 Dynabeads (Gibco, cat. 11161D). Endometrial cancer organoids were harvested, mechanically broken and transferred into each well of a 24-well plate containing 250 μl of T-cell medium. T cells were harvested, counted and combined with organoids at a 25-50:1 (T cells:organoids). Coculture with T cells was performed for 1–3 d at 37°C and 5% CO_2_. Cocultures were imaged in time using an IncuCyte^®^ fast-track scientific discovery system. The imaging settings were as follows: repeat scanning: every 2 h for 72 h; scan type: scratch wound; scan plate: 96-well ImageLock plate. Three images per well were scanned. The supernatants from different groups were collected and LDH Cytotoxicity Assay Kit (Beyotime, C0017) was used to determine the cell viability.

### Statistical analysis

A two-tailed Student’s t test was used to determine the statistical significance of the differences between the means. A p value of < 0.05 was considered statistically significant. We performed statistical analyses using GraphPad Prism version 9.0 (GraphPad software).

## Data Availability

Exon sequencing data from 99 female endometrial cancer (EC) patients at Shanghai First Maternal and Child Health Hospital) can be accessed at NODE: http://www.biosino.org/node (accession no: OEP001202). The remaining data supporting the conclusions of this article will be made available by the authors, without undue reservation.
