# Supplementary figures and images for: Unveiling immunogenic characteristics and neoantigens in endometrial cancer with POLE hotspot mutations for improved immunotherapy
Source: Front Immunol. 2025 Jan 27;16:1528532. doi: 10.3389/fimmu.2025.1528532 (PMC11808158; doi:10.3389/fimmu.2025.1528532)

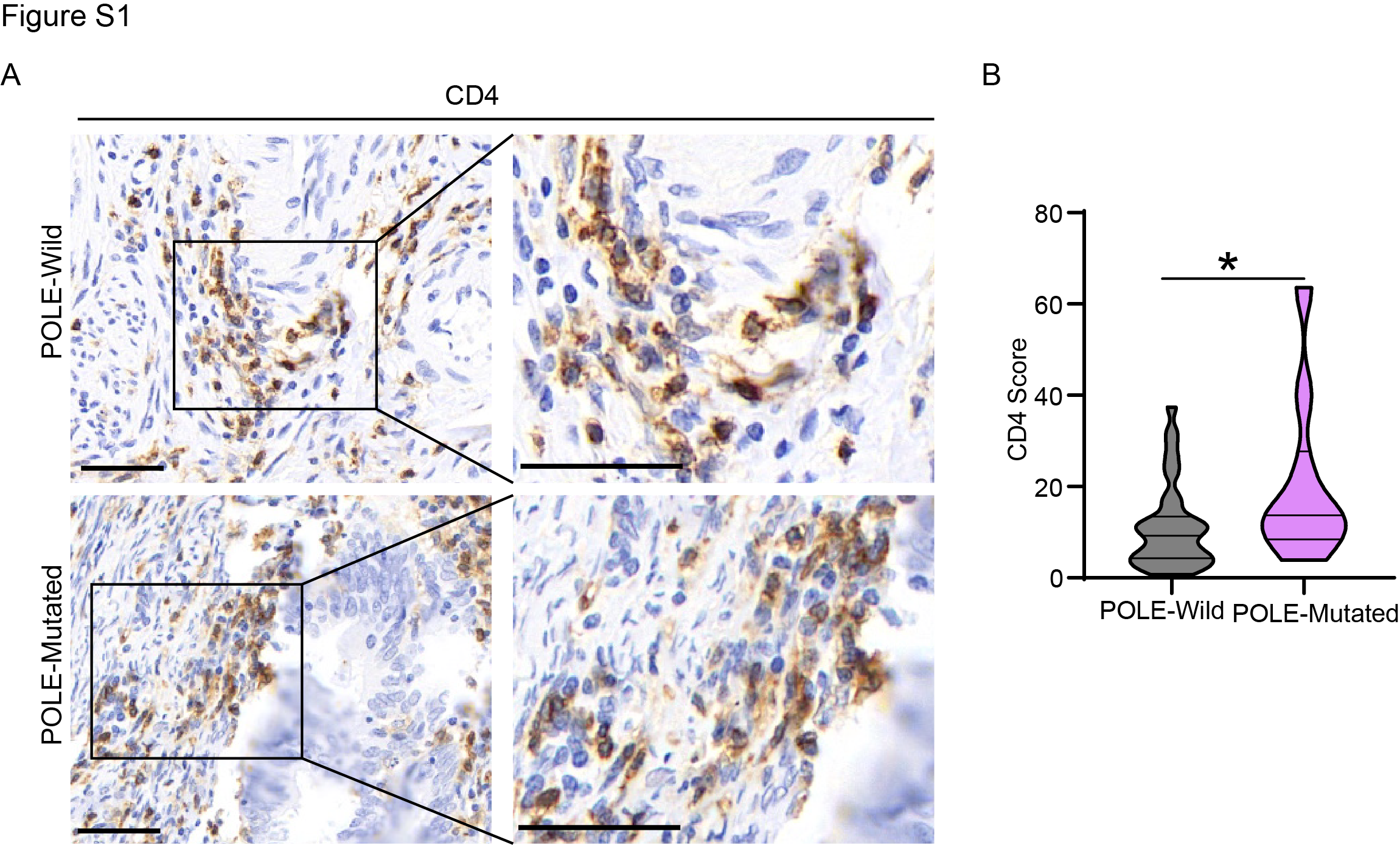

Supplement: Supplementary file 1 [file Image1.tif]

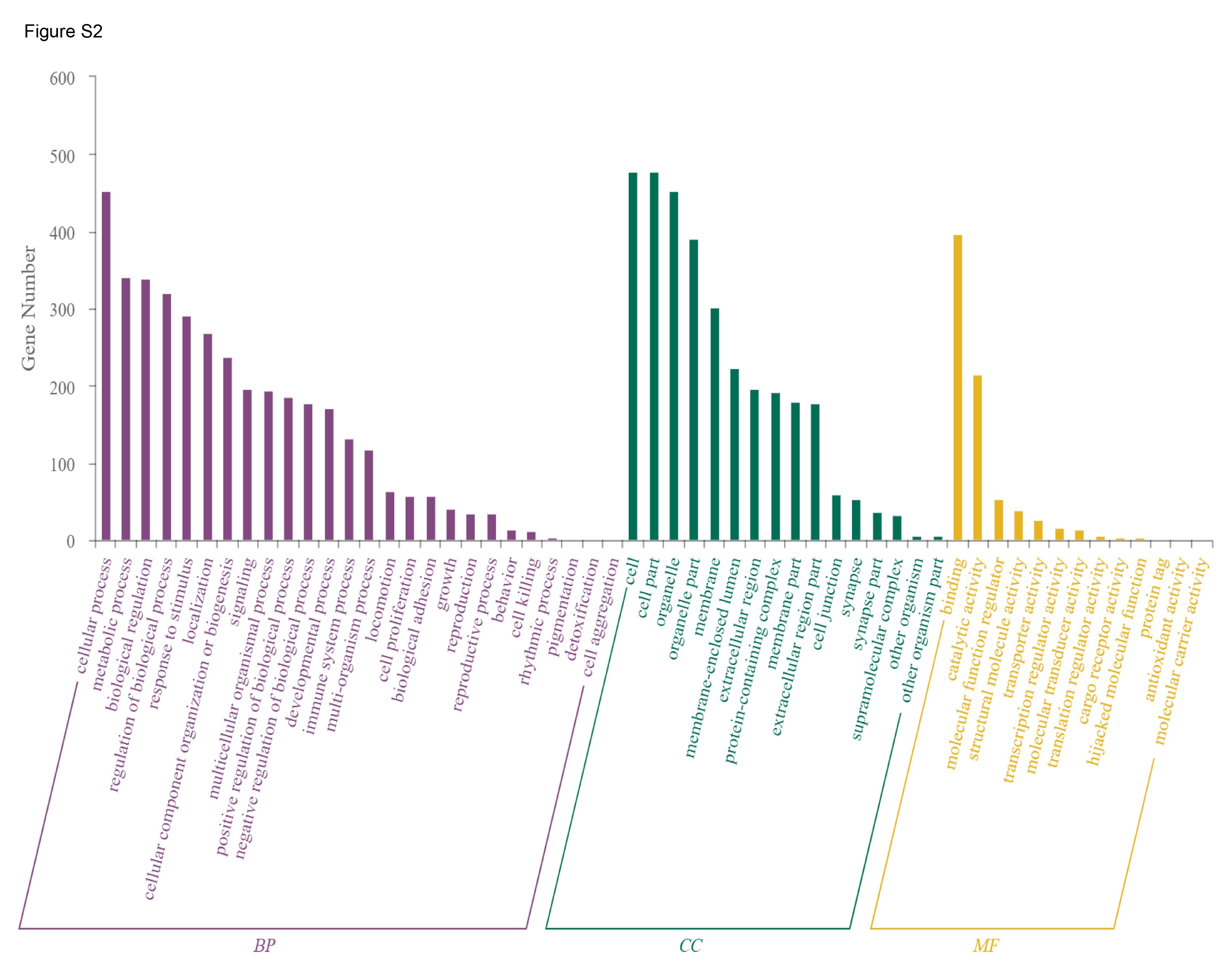

Supplement: Supplementary file 2 [file Image2.tif]
