# Supplementary material for: Unveiling immunogenic characteristics and neoantigens in endometrial cancer with POLE hotspot mutations for improved immunotherapy
Source: Front Immunol. 2025 Jan 27;16:1528532. doi: 10.3389/fimmu.2025.1528532 (PMC11808158; doi:10.3389/fimmu.2025.1528532)
Supplement: Supplementary file 5 [file Table3.docx]

**
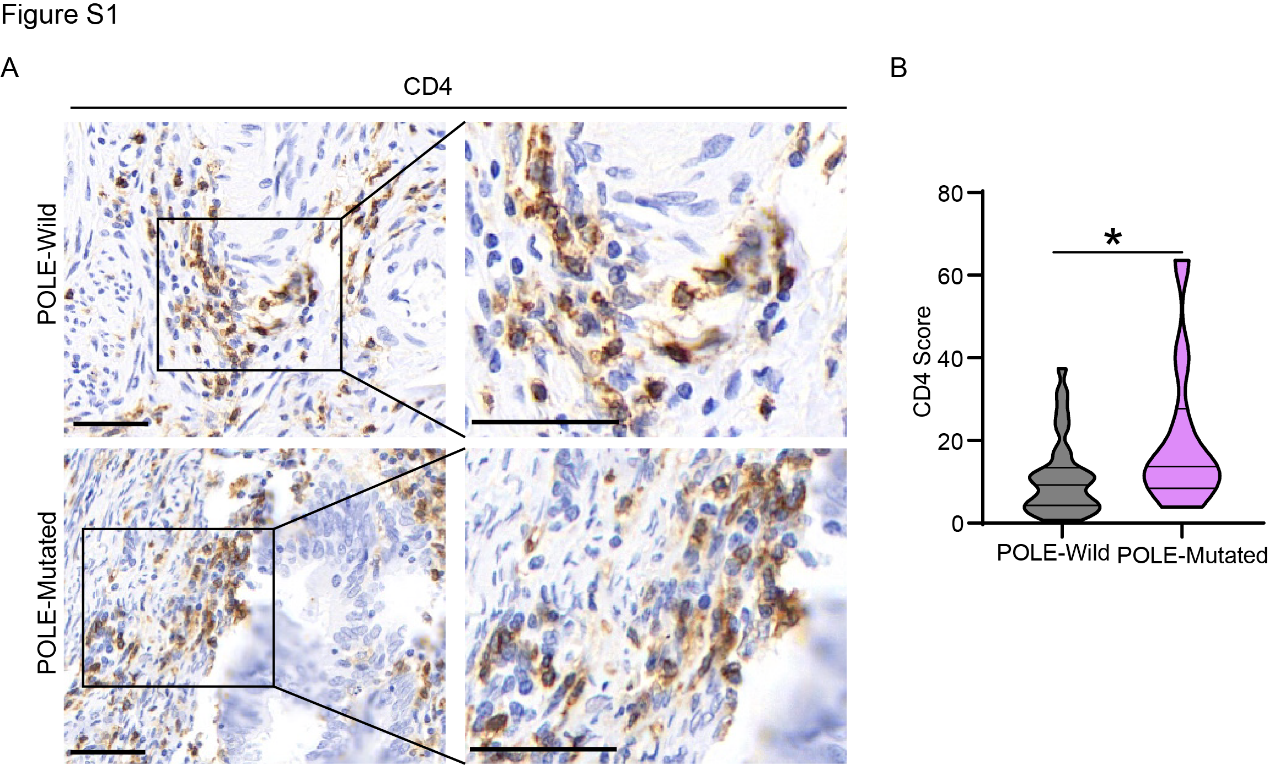
**

**Figure S1. CD4^+^ T cells in POLE-WT and POLE-mutant EC.** (A) Representative images of immunohistochemical staining for CD4 in POLE control and POLE-mutant samples. Scale bar=50μm. (B) Comparison of the CD4 score of immunohistochemical staining between POLE mutant versus wild-type EC patients. Data from 99 EC patients from Shanghai First Maternal and Child Health Hospital. * p<0.05.

**
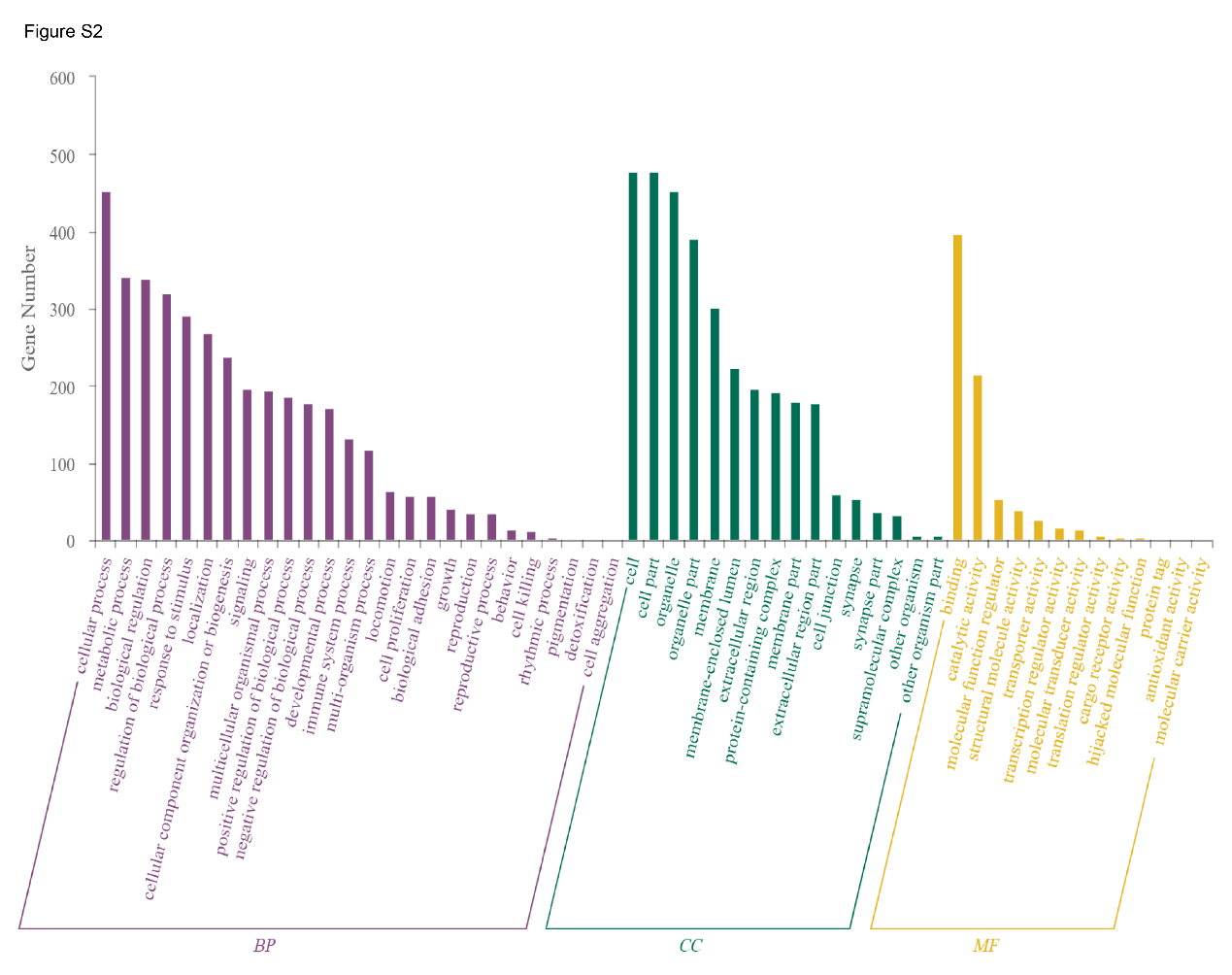
**

**Figure S2. Proteomic GO enrichment analysis of the** **POLE^P286R^ group cell culture supernatant.** The results of GO enrichment analysis showed that the POLE^P286R^ group was active in cell development and metabolic regulation.

**Table S1. Screening of high-frequency POLE mutation loci in endometrial cancer.** Mutation information from clinical samples and public data sources was intersected to obtain information on 91 shared mutants containing the two POLE hotspot mutations, namely, POLE^V411L^ and POLE^P286R^.

**Table S2. Tumor neoplastic antigen prediction.** The software predicted three high-frequency MHC class I molecules (HLA-A*02:01, HLA-A*11:01, HLA-A*24:02) in the Chinese population and determined the binding relationship between HLA and mutant antigen peptide chains. %rank<0.5, the short peptide is considered to bind strongly to MHC-I class molecules; 0.5<%rank<2, the short peptide binds weakly to MHC-I class molecules; %rank>2, the peptide cannot bind to MHC-I class molecules. If the predicted peptide binds more strongly to MHC class I molecules, it is more likely to be a neo-antigenic epitope.

**Table S3. Primers Used for Quantitative PCR**

| Primers | Sequence(5’-3') |
| --- | --- |
| POLE^P286R^-F | TGGTACAATGTCAGATACCGAGG |
| POLE^P286R^-R | CTCATCGGGTTCATTGAAGACAC |
| HLA-A*11:01_1-F | CTCATCGGGTTCATTGAAGACAC |
| HLA-A*11:01_1-R | TGCCAGGTCAGTGTGATCTC |
| HLA-A*11:01_2-F | AGGCTGGTGTCTGGGTTCTGTG |
| HLA-A*11:01_2-R | CCCATCCCGCTGCCAGGT |
| HLA-A*11:01_3-F | CTACCCTGCGGAGATCACACTGAC |
| HLA-A*11:01_3-R | TCCCCTGTGACTTGTGACTGCTG |
| HLA-A*11:01_4-F | CCCTGGGTCTGCAGTCACACAT |
| HLA-A*11:01_4-R | GCACTGTCACTGCCTGGGGTA |

**Table S3. Primers used for quantitative PCR.** The primers (forward and reverse) for the POLE^P286R^ mutation and HLA-A*11:01 were designed. Detailed primer sequences are shown in Supplementary Table S1.
